# A Notable Contest

**Published:** 1916-10-21

**Authors:** 


### The Hospital

The Workers' Newspaper of Administrative Medicine and Institutional Life, Administration, National Insurance and Health.

No. 1585, Vol. LXI. SATURDAY, OCTOBER 21, 1916. PRICE ONE PENNY.

### A NOTABLE CONTEST.

It is curious that while in some departments of medicine the most rigorous canons of evidence are applied and enforced, and no doctrine has a chance of acceptance unless it is supported by flawless proof, in other departments the wildest and most baseless speculations are accepted as gospel without any attempt on the part of their supporters to adduce proof or even evidence. The one set of investigators pursues the method of Bacon, of Harvey, of Newton, and of Darwin; the other pursues the method of Roscellinus, of Lilly, of Paracelsus, and of the great Sequah. No bacteriologist would admit that a given disease was due to a certain bacterium unless that bacterium was found in every case of the disease, unless the bacterium could be identified, isolated, and obtained in pure culture, and unless the inoculation of the pure culture was followed by the occurrence of the disease. Nor would even this last test be regarded as conclusive unless it had been shown, by the employment of controls, that the inoculation alone could be considered the cause. Yet there are those who practise in mental diseases who advance, as causes of these diseases, the wildest speculations, the most baseless surmises, the most futile guesses, and these speculations, surmises, and guesses are received and adopted with as much gravity and as complete faith as the rigorous proofs of the bacteriologist.

Every year or two sees a new fashion in the attribution of causes to mental disease, and one fashion in causes follows another with as little reason and as much caprice as in women's dress. Anything that is being talked about, anything that is in the air will do for a new cause of madness. The sufferers from delusion of persecution drag into their delusion any new discovery that is novel, that is mysterious, that is striking, or that happens to be talked about. First, their persecutions were due to steam; then when electricity came to the fore they were persecuted by electricity; when mesmerism was fashionable they were persecuted by mesmerism; when the Röntgen rays were discovered they formed a new means of persecution; wireless telegraphy followed, and as soon as it was discovered was found to be part of the plot; and nowadays the paranoiac is persecuted by suffocating gases and T.N.T. It is much the same with those who have the care of paranoiacs and other madmen. They used to attribute madness to sexual excess, and especially to masturbation. There was no evidence at all that these practices were causes of madness, and much reason to suppose they could not be causes of it; but in this region of medicine evidence and reason were never regarded and never considered. When bacteria were discovered it was assumed without question, and equally without reason and without evidence, that insanity was due to a microbe. When it was found that much of the mischief of microbic diseases was due to the toxins secreted by the microbes, at once madness was attributed to toxins of microbic origin. Then came intestinal stasis and alimentary toxæmia, fertile causes of disease both of them, and madness was added to the long list of diseases thus caused. Now that the immense and mysterious powers of the secretions of the ductless glands are being revealed, they are, of course, impressed into the service, and are declared to cause insanity by their excess or defect.

It is a familiar fact that when the paranoiac is at a loss for some new discovery to which he can attribute his persecution, he invents one for himself, he invents some agent so recondite and mysterious that no one but himself has ever heard of it, and he names it accordingly by some new appellation of his own invention. He is persecuted by cubical feet, by hypophones, by infernal traces mystery, or by some other meaningless invention of his own for which his settled and profound conviction takes the place of evidence and of reason. And the physician for mental diseases is not to be outdone by his patient. He shows himself not inferior to his patient in fertility and extravagance of invention. "What," he says in effect to his paranoiac patient, "what is your invention in comparison with mine? Do you suppose that I shall allow a lunatic to beat me in a field that I have made peculiarly my own? Do not flatter yourself that you have any chance against me when it comes to a contest in extravagance and irrationality. You may have your cubical feet and your hypophones, but I go one better with psycho-analysis. There may, for aught I know, be some evidence for the existence of hypophones or infernal traces mystery, but I defy you to find any evidence in favour of psycho-analysis. You may have some glimmering of reason at the foundation of your belief: I defy you to find any at the bottom of mine!" It is impossible to withhold the palm from the physician. He has met his patient fairly upon common ground and he has beaten him out and out. All honour to the victor in such a noble contest!

